# Legionnaires’ disease: overview of the situation concerning notification in Wallonia (Belgium) in 2012, a retrospective descriptive study based on a capture-recapture method

**DOI:** 10.1186/2049-3258-73-2

**Published:** 2015-01-12

**Authors:** Stéphanie Jacquinet, Olivier Denis, Filomena Valente Soares, Carole Schirvel

**Affiliations:** Infectious diseases surveillance, Fédération Wallonie-Bruxelles, Belgique, Direction générale de la santé, cellule de surveillance des maladies infectieuses, Boulevard Léopold II, 44, 1080 Bruxelles, Belgique; department of microbiology, hôpital Erasme, Centre National de Référence des Légionelles, 1070 Bruxelles, Belgique; Ecole de Santé Publique, Université Libre de Bruxelles, Campus Erasme - CP598 808 route de Lennik, 1070 Bruxelles, Belgique

**Keywords:** Legionnaires’ disease, Surveillance, Belgium

## Abstract

**Background:**

Legionnaires’ disease is a severe form of pneumonia, and although public health medical inspectors must be notified, it is often under-reported. The objectives of this study were to determine the completeness rate of notification of Legionnaires’ disease and to estimate the incidence of this disease in Wallonia, the southern part of Belgium, in 2012.

**Method:**

This retrospective, transversal descriptive study was based on a capture-recapture method using two sources. An estimation of the total number of Legionnaires’ disease cases was calculated using Chapman and Seber’s estimators for small numbers, thereby allowing us to estimate the real incidence of this disease in Wallonia as well as the completeness rate of notification.

**Results:**

The total number of estimated *Legionella* cases for 2012 was 45 (IC 95%:41–48) and the completeness rate was 65% (IC 95%:61-70%). The estimated incidence of Legionnaires’ disease in Wallonia was 1.27/100,000 inhabitants.

**Conclusions:**

The notification rate of *Legionella* must be improved in Wallonia. Doctors should be made aware of the importance of diagnosing and reporting Legionnaires’ disease.

## Français

**Introduction:**

Malgré une mortalité et morbidité non négligeables, la maladie du légionnaire est une maladie à déclaration obligatoire auprès des médecins inspecteurs qui est fréquemment sous rapportée. Les objectifs de cette étude furent le calcul de l’exhaustivité de la déclaration obligatoire de la maladie du légionnaire et l’estimation de son incidence en Wallonie, la partie sud de la Belgique, pour l’année 2012.

**Méthodologie:**

Cette étude descriptive transversale rétrospective s’est basée sur une méthode capture-recapture à 2 sources. Une estimation du nombre total de cas de maladie du légionnaire a été réalisée en utilisant les estimateurs de Chapman et Seber pour effectifs faibles. Ensuite, l’incidence réelle de la maladie du légionnaire en Wallonie ainsi que l’exhaustivité de la déclaration ont pu être calculées.

**Résultats:**

Le nombre total de cas estimés de maladie du légionnaire pour l’année 2012 est de 45 (IC 95% : 41–48) et le taux d’exhaustivité de la déclaration obligatoire est de 65% (IC 95% : 61-70%). L’incidence estimée de maladie du légionnaire en région wallonne est de 1,27/100.000 habitants.

**Conclusion:**

Le taux de notification de la maladie du légionnaire doit être amélioré en Wallonie. Les médecins devraient être mieux sensibilisés à la déclaration et au diagnostic de la maladie du légionnaire.

## Background

Legionnaires’ disease is an obstructive pulmonary disease caused by *Legionella* (gram-negative bacteria). *Legionella pneumophila* species accounts for 90% of Legionnaires’ disease cases and included 15 different serogroups, among which we find serogroup 1, which causes 70% to 90% of *Legionella* cases for which a bacteria has been isolated [1, 2].

*Legionella* is naturally present in freshwater but also grows in the presence of different favouring factors, such as temperatures between 25° and 42°C, the presence of nutrients and protozoa and the possibility to form a biofilm when scale, corrosion and stagnation are present in the water system [3]. Human infection occurs through the inhalation of micro-drops containing bacteria. These droplets can be generated by showers, hot tubes, cooling towers, indoor fountains, humidifiers, respiratory devices and nebulizers [3].

Legionnaires’ disease is a serious disease which accounts for 0.5 to 10% of hospitalizations of community-acquired pneumonia [3]. Moreover, it is associated with a serious case fatality rate (10%) [4]. It can occur in sporadic cases or outbreaks, affecting many people [1].

Legionnaires’ disease mainly affects people with some risk factors: the elderly, males, smokers, the presence of a chronic heart or lung disease, diabetes, immunosuppression, end-stage renal failure, neoplasia and hematological malignancy [1–3].

**Figure 1:**
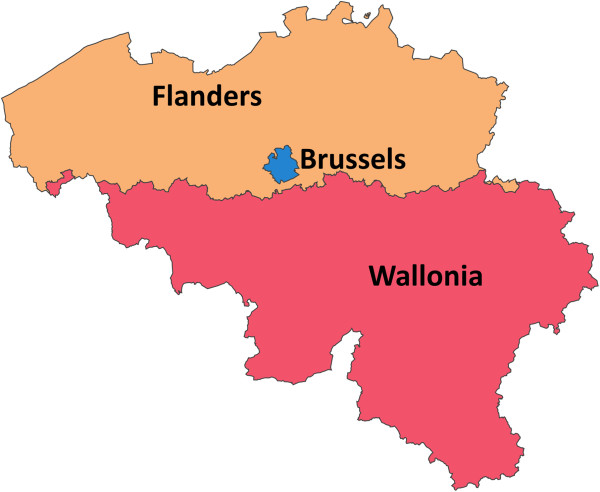
**The 3 regions of Belgium.**

In Belgium, the physician is obliged to notify public health medical inspectors cases of Legionnaires' disease as is the case in many European countries. The infectious disease notification is a tri-region responsibility (Figure 1). This notification allows the public health medical inspectors to conduct an environmental enquiry in order to determine the most probable source of infection and to implement control measures to avoid contaminating other people. Unfortunately, this disease is frequently under-diagnosed and under-reported [2] thereby hampering the detection of clusters of Legionnaires’ disease patients and hindering investigation into the possible source of *Legionella* infections. The completeness of notification of this disease has never been quantified, so the scale of under-reporting is unknown. Moreover, the real incidence of Legionnaires’ disease in Wallonia has never been calculated and, if it were, this knowledge would make it possible to measure the burden of the disease on the Walloon population.

The purposes of this study were to calculate the completeness of notification of Legionnaires' disease and to measure its incidence in Wallonia in 2012.

## Methods

It is a retrospective cross-sectional descriptive study using a capture-recapture method with two sources. The capture-recapture method allows a cross referencing of several diagnostic sources of disease to find common cases and estimate the number of cases that are not found in either of the sources. The total number of cases and the completeness rate of each source can then be calculated. Through the use of two sources, a contingency table can be created (Table 1) where the total number of cases not observed in both sources (X) can be expressed as X = (b*c)/a.

**Table 1:** **Contingency table of capture recapture study with 2 sources**

	Source A			Total register B
		Observed	Not observed	
**Source B**	**Observed**	a	b	N_B_
	**Not observed**	c	X	
**Total register A**		N_A_		N

Because of the low number of cases in this study, Chapman’s estimator [5] was used to calculate the total number of Legionnaires' disease cases in Wallonia (N) and the variance for N (var N) was calculated with Seber’s estimator [6]:


To calculate the confidence interval of 95% of N (IC95%), the following formula was used:


Then, the completeness rate of the source (E) can be calculated. For example, for source A (Table 1), E = NA/N.

The study targeted patients living in Wallonia whose symptoms started between the 1^st^ January 2012 and 31^st^ December 2012.

### Case definition

The study included confirmed or probable Legionnaires' disease cases according to the case definition of the ECDC [7].

### Data collection

The first source for this study was notification to the Walloon public health medical inspectors in 2012.

The second source was the “acute hospitals” in Wallonia [8]. Specialized chronic-care facilities and psychiatric hospitals were excluded from the study. Given that it is necessary to conduct a laboratory test to diagnose Legionnaires' disease, microbiologists from the different Walloon hospital laboratories were contacted. An information letter describing the study and its methodology was sent to microbiologists early in January 2013. The microbiologists in the concerned laboratories were then contacted by telephone during January. Forty-two laboratories carrying out analyses for the 57 acute hospitals of Wallonia were contacted.

### Capture recapture and data analyses

For both sources, a structured questionnaire was used and specific data was collected: the first letter of the surname, the forename, gender, date of birth, postcode, and laboratory tests that was/were used for the diagnosis and the X-ray results. This data was necessary to detect duplication and to ensure those cases corresponded to the definition of the case. Then, both data sources were compared to identify the common cases and those that were present in each of the sources.

Afterwards, the completeness rate of notification was calculated as well as the incidence of Legionnaires' disease (the average in the population figures on 1^st^ January 2012 and on 1^st^ January 2013 [9]) in Wallonia.

The total number of cases and the incidence were calculated using Excel software (12.0 version).

### Ethical consideration

This study was approved by the ethical committee of Erasmus Hospital in Brussels.

## Results

**Figure 2:**
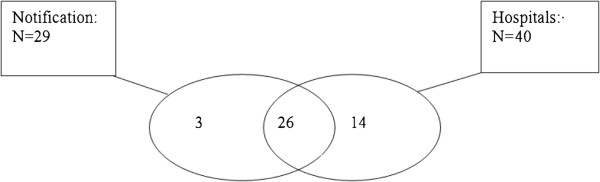
**Distribution of Legionnaires’ disease cases according to the 2 sources.**

The response rate from the hospital laboratories amounted to 100%. One case that was reported by a laboratory was excluded because the patient’s place of residence was not in Wallonia.Twenty-nine cases were found through notification, 40 with the acute hospitals and 26 common cases were identified by both sources (Figure 2). Fourteen cases were observed from the source “notification” and 3 cases that were reported to the public health inspectors were not taken into account during the recapture from the hospital laboratories (Figure 2).

Following the use of Chapman’s estimators, it appears that two cases were identified by none of the sources and that the total number of estimated cases of Legionnaires' disease for 2012 amounted to 45 (IC 95%: 41–48) (Table 2).

**Table 2:** **Contingency table of the capture-recapture study of Legionnaires’ disease cases diagnosed in 2012**

	Source A: notification			Total register B
		Observed	Not observed	
**Source B:**	**Observed**	26	14	40
**Acute hospitals**	**Not observed**	3	2	
**Total register A**		29		45

The completeness rate of notification for Legionnaires' disease cases was 65% (IC 95%: 61%-70%).

The estimated incidence of Legionnaires’ disease in Wallonia is thus 12.7 per million inhabitants.

## Discussion

The estimated incidence of Legionnaires' disease in Wallonia in 2012 (12.7 per million inhabitants) was quite close to the European average of 12.4 per million inhabitants in 2010 [4] and that of the United States of 11.5 per million inhabitants [10]. However, the incidence was very far from the one expected in Europe by the ECDC. Two estimations of the expected incidence of Legionnaires' disease in Europe have been made by the ECDC to-date. The first estimation was 20 per million inhabitants [11] by choosing Denmark as a reference because it is a small country whose incidence of Legionnaires’ disease has remained constant for several years and which conducts a high number of diagnostic tests for Legionnaires' disease. A second incidence was calculated theoretically in 2009 [12] (starting from a theoretical incidence of pneumonia and by estimating that the proportion of pneumonia due to *Legionella* is 4%). It estimates the real incidence of Legionnaires' disease at 103 per million inhabitants.

An explanation for the low incidence could be the under-diagnosis of the disease and there are various reasons that are connected to it. A lack of sensitivity in some diagnostic methods and the very frequent use of urine testing which only detects *L.pneumophila* of serogroup 1 is the first cause of under-diagnosis [13]. Legionnaires' disease is known to cause severe cases of pneumonia: a moderate clinical picture of lung infection doesn’t always lead to the diagnosis of Legionnaires' disease [13] and pneumonia reported in Wallonia in 2012 was diagnosed exclusively among hospitalized patients.

Another cause of under-diagnosis is the immediate use of empirical antibiotic therapy for pneumonia especially in severe cases. This treatment is, among others, justified for Legionnaires' disease because a rapid response diminishes the risk of mortality [2]. If the antibiotic is effective against legionella, the patient will recover and the medical doctor won't see the necessity in searching for the cause of the pneumonia [13].

The completeness rate of notification in Wallonia is estimated at 65%. Other European countries conducted capture-recapture studies in order to measure their completeness: in 2002 it was estimated at 78.6% in Italy [14], in France in 2010 at 88.5% [15] and in the Netherlands in 2000–2001 at 42.1% [16]. European rates are rather heterogeneous and Wallonia’s one shows there could be a significant number of Legionnaires' disease cases for which no investigation was conducted to determine the source of contamination and for which no control measures were taken. Moreover, under-reporting makes it impossible to evaluate the real impact of the disease on a public health level.

The reasons for under-reporting diseases with mandatory declaration are numerous: a lack of knowledge concerning the list of diseases that must be reported, the reporting method or even the existence of notification are the main causes [17, 18]. Among doctors that are well informed about the notification, the belief that another colleague will report it, a lack of time and the cumbersome procedure are also mentioned. Some doctors also refuse to notify (the investigation in France in 2005 shows that 16% of informed doctors refused to report) [17]. This refusal can probably be explained by a lack of awareness of the importance of notification on public health.

In Wallonia, no study has yet been conducted to analyze the reasons for under-reporting and a great number of the reasons mentioned above should probably be taken into account. However, the reporting procedure is rather simple and can be done in several ways, among others, by an internet interface, and in all cases by straightforward reporting to the public health medical inspector without intermediaries.

Concerning the methodological aspects, the use of Walloon hospitals as a second source makes it possible to capture only hospitalized cases. But, Legionnaires' disease can also present as a moderate case of pneumonia [3] and thus, occur among non-hospitalized patients, which could be interpreted as a methodological weakness in this study. However, Legionnaires’ disease is unlikely to be diagnosed among non-hospitalized patients because the recommendations in that matter are not geared towards research of etiologic pathogens among non-hospitalized patients [19]. In France, between 1998 and 2008 only 2% of reported *Legionella* cases were not hospitalized [20], which still supports the hypothesis that very few cases are diagnosed on an out-patient basis.

Given that, in this study, laboratories were obliged to report all results, including those sent to a private laboratory, all hospitalized cases were thus, in principle, identified. However, microbiologists, who don’t usually report Legionnaires' disease cases, may have omitted to notify some cases during the capture-recapture investigation. Moreover, as the data retrieval in laboratories isn’t always computerized, some cases may not have been reported.

Brussels and Flemish hospitals were not included in the second source as some patients residing in Wallonia and hospitalized in these regions may not have been identified. However, for 2012, no Walloon case was declared by Flanders and only one Walloon case was declared by a Brussels hospital.

The response rate of 100% from hospitals leads us to conclude that a vast majority of Legionnaires' disease cases among hospitalized patients were identified.

Most of the assumptions of the capture-recapture study have been respected: the case definition was the same for both sources, the cases appeared during the same period and in the same geographical zone and all the common cases were fully identified [21, 22]. However, in human conditions, limitations are described for the following assumptions: close population, homogeneous population and independence of the sources [23, 24]. In this study, a dependence between sources is possible because the microbiologists used to produce the second list were subject to mandatory declaration. It would then be a positive dependence which would lead to an underestimation of the number of Legionnaires' disease cases and thus an overestimation of the completeness [21, 23].

It would have been preferable to conduct that capture-recapture study with three sources to allow us to check the independence of the sources [21, 22]. However, the use of sentinel laboratories or of National Reference Centers as a third source was not possible because it would have lead to a dependence with the first two sources. The Hospital Discharge Record database could, however, have been a third source. They have already been used for that type of study by different countries, among which the Netherlands [16] and Italy [14], by researching the cause of certain types of pneumonia according to the ICD-9 CM. The problem we would then be confronted with would be the difference in the case definition.

## Conclusion

Given the probable under-diagnosis of this disease in Wallonia and the fact that the completeness rate of notification only reaches 65%, it remains very important to raise awareness amongst doctors of the diagnosis of Legionnaires' disease and to continue to inform them on the necessity and the importance to notify the public health authorities and the usefulness of doing so.

## Endnotes

This category of hospitals refers to general hospitals (including university hospitals) with the exception of specialized and geriatric clinical establishments.

## Abbreviations

ECDC: European Centre for Diseases Prevention and Control
ICD-9 CM: International Statistical Classification of Diseases Ninth Revision Clinical Modification.
